# A novel variant in the neutrophil cytosolic factor 2 (*NCF2*) gene results in severe disseminated BCG infectious disease: A clinical report and literature review

**DOI:** 10.1002/mgg3.1237

**Published:** 2020-04-12

**Authors:** Suzan A. AlKhater, Caroline Deswarte, Jean‐Laurent Casanova, Jacinta Bustamante

**Affiliations:** ^1^ Department of Pediatrics King Fahad Hospital of University, Al-Khobar Imam Abdulrahman Bin Faisal University College of Medicine Dammam Saudi Arabia; ^2^ Laboratory of Human Genetics of Infectious Diseases Necker Branch INSERM U1163 Necker Hospital for Sick Children Paris France; ^3^ Paris University Imagine Institute Paris France; ^4^ St Giles Laboratory of Human Genetics of Infectious Diseases Rockefeller Branch The Rockefeller University New York NY USA; ^5^ Howard Hughes Medical Institute New York NY USA; ^6^ Pediatric Hematology and Immunology Unit Assistance Publique‐Hôpitaux de Paris Necker Hospital for Sick Children Paris France; ^7^ Center for the Study of Primary Immunodeficiencies Necker Hospital for Sick Children AP‐HP Paris France

**Keywords:** BCG vaccine, chronic granulomatous disease, *NCF2* gene, p67*^phox^* protein, primary immunodeficiency disorders

## Abstract

**Background:**

Chronic granulomatous disease (CGD) is a rare primary immunodeficiency disorder (PID) affecting NADPH oxidase activity. The rarest form of the disease is considered to be caused by *NCF2* gene bi‐allelic variant. Here, we report the clinical and molecular characterization of a patient presenting with early‐onset severe disease due to bi‐allelic *NCF2* variant.

**Methods:**

Gene mutational analysis was performed by whole‐exome and Sanger sequencing.

**Results:**

The patient presented with a history of fever and rash since the age of 1 month, followed by destructive osteomyelitis and necrotizing lymphadenopathy. The patient received the Bacillus Calmette‐Guérin (BCG) vaccine at birth; she was subsequently diagnosed with disseminated BCG infection. Whole‐exome sequencing identified a private (unreported) homozygous variant in *NCF2* (c.290C > A) that results in a nonconservative change, p.Ala97Asp, in the p67*^phox^* protein. The variant is located in the third helix of the TRP domain, which is crucial for the binding of GTPase RAC2 to the NADPH oxidase complex.

**Conclusion:**

We identified a novel *NCF2* variant located in the region interacting with RAC2 that is linked to a severe and early CGD phenotype in the setting of disseminated BCG infection. Our findings support postponing BCG vaccination until 6–12 months of age and after PID assessment.

## Graphical abstract

Chronic granulomatous disease (CGD) is a rare primary immunodeficiency disorder (PID) affecting NADPH oxidase activity. Bi‐allelic mutation of the NCF2 gene is considered the rarest form of the disease. Here, we report the clinical and molecular characterization of a patient presenting with early onset severe disseminated BCG infection due to bi‐allelic NCF2 mutation.

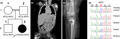

## 1 INTRODUCTION

Primary immunodeficiency disorders (PIDs) constitute a diverse group of rare hereditary disorders that affect the immune system and result in impaired immune responses and a predisposition to severe infections (Alkhater, 2009). Among PIDs, chronic granulomatous disease (CGD) is caused by the inability of phagocytes to create an effective oxidative burst, which enables cells to kill intracellular parasites using reactive oxygen species (ROS) (de Oliveira‐Junior, Bustamante, Newburger, & Condino‐Neto, 2011; Thomas, 2017). The underlying defect is caused by pathogenic variants in the genes that encode the subunits of the nicotinamide adenine dinucleotide phosphate (NADPH) oxidase complex. This complex is a vital component of the innate immune system and is mostly active in phagocytes (de Oliveira‐Junior et al., 2011). Variants in the *CYBA*, *CYBB*, *NCF1*, *NCF2* (OMIM *608,515), *NCF4,* and *CYBC1* genes, which code for the six components of this enzyme complex, lead to CGD (Roos et al., 2010). Hemizygous variants in the *CYBB* gene, which encodes the gp91*^phox^* subunit of the NADPH oxidase complex, lead to X‐linked recessive (XL) CGD, while bi‐allelic variants in the *CYBA*, *NCF1*, *NCF2*, *NCF4,* and *CYBC1* genes, which encode the p22*^phox^*, p47*^phox^*, p67*^phox^*, p40*^phox^*, and EROS subunits, respectively, lead to different forms of autosomal recessive (AR) disease (Arnadottir et al., 2018; Chiriaco, Salfa, Di Matteo, Rossi, & Finocchi, 2016; Roos et al., 2010; Thomas et al., 2017). CGD is characterized by recurrent bacterial, including mycobacterial, and fungal infections, resulting in granulomas, episodes of fever, rash, and other symptoms, such as colitis (Roos et al., 2010). Susceptibility to *Mycobacterium* infections, including *Mycobacterium tuberculosis*, nontuberculosis *Mycobacterium*, and Bacillus Calmette‐Guérin (BCG), may be the initial presentation (Conti et al., 2016). The defect in NADPH oxidase activity renders the phagocytes unable to kill intracellular pathogens. This carries a particular risk for infants with CGD who have been received the BCG vaccine (Conti et al., 2016). Other disorders that carry a similar risk include patients with Mendelian susceptibility to mycobacterial disease (MSMD), a rare genetic disorder affecting innate immunity and resulting in susceptibility to weak mycobacterial pathogens, including environmental mycobacteria and the BCG vaccine (de Beaucoudrey et al., 2010; Bustamante, Boisson‐Dupuis, Abel, & Casanova, 2014; Casanova, 2015; Prando et al., 2013). The vaccine comprises live attenuated *M. bovis* and is the only readily available vaccine for tuberculosis (Trunz, Fine, & Dye, 2006). However, the vaccine is associated with severe adverse events in susceptible patients (Bukhari et al., 2016). Here, we report the presentation, clinical features, and genetic results of a patient with CGD presenting with severe disseminated BCG infection (BCG‐osis). Comparisons to previously reported variants in the isolated gene are discussed.

## 2 MATERIALS AND METHODS

### 2.1 Ethical compliance

All procedures performed in this study were in accordance with the ethical standards of the institutional ethics committee at King Fahad Hospital of University, Al‐Khobar, Saudi Arabia (Institutional Review Board (IRB) number IRB‐2019–01–123), and with the 1964 Helsinki declaration and its later amendments or comparable ethical standards. Informed consent was obtained from all individual participants included in the study. Additional informed consent was obtained for publication from all individual participants for whom identifying information is included in this article.

A dihydrorhodamine 123 (DHR) flow cytometry test was used to detect reduced superoxide production by stimulated neutrophils as previously described (Vowells, Sekhsaria, Malech, Shalit, & Fleisher, 1995). The patient's neutrophil oxidative index (NOI) was determined by calculating the ratio of the mean fluorescence of stimulated cells to that of background control cells. A laboratory reference value of NOI < 87 is consistent with a diagnosis of CGD. After obtaining consent from parents to perform the genetic diagnostic assay, a 5‐ml blood sample was obtained from both parents and the child and dissolved in heparin. Genomic DNA (gDNA) was extracted for whole‐exome and Sanger sequencing.

## 3 RESULTS

**Figure 1:**
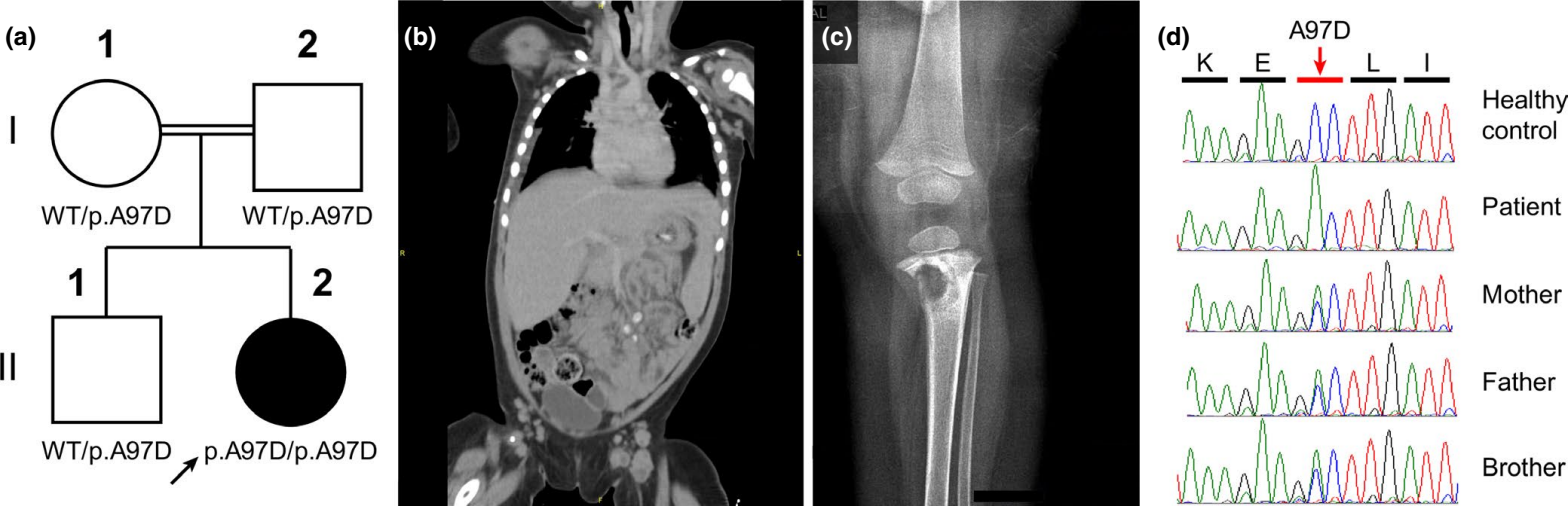
Deficiency of p67phox in a child with disseminated BCG infection. (a) Pedigree of kindred, showing the index case, indicated with an arrow, and her brother and parents; each generation is designated by a Roman numeral (I‐II). (b) Abdominal CT scan showing hepatosplenomegaly, intra‐abdominal calcified mesenteric, and para‐aortic lymph nodes. (c) Anteroposterior radiograph of the patient's left knee demonstrating a well‐circumscribed lucent lesion in the tibial metaphysis and posterior cortical disruption. (d) Electropherogram showing the position of the variant (c.290C > A; p.Ala97Asp; NM_001127651.2), in the *NCF2* gene in a healthy control and the family members

The patient was born full term weighing 3.8 kg via Cesarean section due to failure of labor progression and was immediately admitted to the neonatal intensive care unit for 10 days for observation due to a severe erythematous rash that completely resolved without intervention. The parents are first‐degree cousins (Figure 1a). The patient is the second child in a family with no prior history of immune deficiency. She received the BCG vaccination in her left deltoid muscle on day 1 of life, which is standard practice in the Kingdom of Saudi Arabia. At the age of 1 month, the infant developed recurrent fever and an extensive rash that required multiple hospital admissions. Despite extensive investigations for a focal point, none was found, and initial evaluation of infectious etiologies did not reveal any pathogens. In addition, she exhibited a poor response to various antimicrobial therapies. The febrile episodes subsided at the age of 4 months but have recently recurred. At the age of 9 months, the parents noticed poor healing of the BCG vaccine scar along with ulceration and oozing at the site of injection. The patient developed left axillary lymphadenitis and an abscess, for which an incision and drainage were performed at an outside hospital. No supporting culture reports were available. Her parents also reported diarrhea 4–5 times per day with no blood or mucus. During examination, the patient was found to be irritable with crusted pustular lesions extensively distributed over her face, trunk, and extremities. Swelling and erythema of the lips with severe gingivostomatitis and multiple oral ulcerative lesions on the lips, gums, and palate were observed. Moreover, a left axillary wound from the previous incision and a drainage scar were healing poorly, with an underlying palpable lymph node. The patient had hepatosplenomegaly, and bilateral inguinal glands were palpable. She had tenderness of her left lower limb, and a warm cystic lesion was palpable on the back of her left knee. Perineum examination revealed thrush and an anal skin tag. The investigation included complete blood counts (Table 1) and a stool examination, which was positive for occult blood but negative for culture, ova, or parasites. A computed tomography (CT) scan revealed generalized, enlarged, necrotizing lymph nodes, several of which were matted and calcified (Figure 1b). A plain X ray of the left lower limb (Figure 1c) and magnetic resonance imaging (MRI) of the lower limbs revealed a destructive process involving the left tibia associated with a pathological fracture and enlarged, necrotic, lymph nodes in the popliteal fossa, femoral, and inguinal regions. A bone biopsy was not performed because of parental refusal, and efforts to isolate the etiological agent from other sites also failed. Biopsy of the left inguinal lymph node revealed sterile, necrotizing, and granulomatous inflammation. A polymerase chain reaction (PCR) assay did not detect *M. tuberculosis* complex, and the culture was negative. In addition, during her hospital stay, the patient developed respiratory failure and required intubation. Her thoracic CT demonstrated bilateral pulmonary infiltration and bilateral pleural effusion. Based on her presentation, a clinical diagnosis of BCG‐osis was made. Therapy with standard doses of isoniazid, rifampin, ethambutol, and pyrazinamide was initiated. An immunological workup for an underlying PID was performed (Table 1). Lymphocyte phenotyping demonstrated elevated numbers of T and B lymphocyte subsets. A lymphocyte proliferation assay demonstrated a normal response to mitogens and antigens. An evaluation of leukocyte adhesion defects was also performed due to the presentation of severe leukocytosis, periodontitis, and delayed wound healing, but this condition was ruled out based on normal granulocyte expression. A DHR test revealed absence of NADPH oxidase activity in the patient's neutrophils upon PMA activation, and the patient's NOI was < 1. Based on this result, a diagnosis of CGD was made. We performed whole‐exome sequencing of the patient, which revealed a private (unreported) homozygous variant, c.290C > A, in the *NCF2* gene (NM_001127651.2) resulting in the nonconservative change p.Ala97Asp. The variant was verified by Sanger sequencing. The parents and her brother were heterozygous for this variant (Figure 1d). Polyphen2 (probably damaging, score 1 in HumDiv and score 0.998 in HumVar), SIFT (deleterious, score: 0.01), and MutationTaster (disease causing, p‐value: 1) predicted a high in silico impact of the variant. Prophylaxis with cotrimoxazole (trimethoprim 6 mg kg^‐1^ day^‐1^) and itraconazole (5 mg kg^‐1^ day^‐1^) was initiated. The patient showed improvement in her general condition and her respiratory status. In addition, she showed healing of the osteomyelitis and the pathological bone fracture, as confirmed by repeat MRI imaging. She was referred to a transplant center for evaluation for hematopoietic transplantation.

**Table 1:** Laboratory data

Immunological workup	Results	Reference range
Hemoglobin (g/dl)	10.5	11.3–14.1
White blood cells/mm^3^	51,000	5,000–17,000
Neutrophils/mm^3^	29,070	1,000–6,000
Lymphocytes/mm^3^	16,830	4,000–12,000
Monocytes/mm^3^	5,100	200–1,200
Platelets/mm^3^	722,000	140–400
Immunoglobulin levels		
IgA (mg/dl)	245	20–100
IgG (mg/dl)	1,539	453–916
IgM (mg/dl)	243	19–146
IgE (IU/ml)	53.1	≤97
Lymphocyte populations		
CD3/mm^3^	4,298	2,200–4,100
CD4/mm^3^	3,035	1,400–2,800
CD8/mm^3^	1,235	800–1,800
CD19/mm^3^	2,565	700–1,600
CD65/16/mm^3^	928	200–600
Granulocyte expression		
CD11b	100%	
CD18	100%	
CD15s	96%	

## 4 DISCUSSION

CGD is caused by defective NADPH oxidase activity in phagocytes, which renders them unable to kill intracellular pathogens (de Oliveira‐Junior et al., 2011). The patient reported here had an early presentation with severe symptoms. She showed poor healing of the BCG scar, clinical signs of disseminated mycobacterial infectious disease, and laboratory results consistent with CGD. This diagnosis was subsequently confirmed by the absence of NADPH oxidase activity in neutrophils and the detection of the presence of a novel homozygous variant in the *NCF2* gene. Since their first description as the cause for CGD in 1988 (Volpp, Nauseef, & Clark, 1988), variants in the *NCF2* gene, the gene encoding the p67*^phox^* protein of the NADPH oxidase complex, have been found in many different patients (Table 2). Variants in *NCF2* cause one of the rarest forms of the disease and account for 6% of all cases of CGD (Roos et al., 2010). Volpp et al. (1988) were the first to demonstrate that the p67*^phox^* protein was absent in neutrophils of certain patients with AR CGD. The authors described p67*^phox^* as an active neutrophil cytosolic factor that is critical for superoxide production in phagocytic cells. Okamura et al. (1990) then described the role of p67*^phox^* in relation to the NADPH oxidase respiratory burst function and suggested that the protein forms a complex with cytosolic p47*^phox^*. Furthermore, the binding of p67*^phox^* to a small protein, RAC2, a vital GTPase protein, was found to be necessary for the formation of the cytosolic complex, along with p47*^phox^* and the subsequent assembly of the other components of the NADPH enzyme complex (Mizuno et al., 1992). This is mediated via the N‐terminal region of p67*^phox^*, the tetratricopeptide repeat (TRP) domain (Koga et al., 1999). However, de Boer et al. (1994) were the first to report the genetic code for the missing protein, as this group identified a missense variant of G‐233 as the underlying genetic defect for p67*^phox^* deficiency in AR CGD patients. The variant found in our patient had not previously been described; however, it is in a region similar to that described by Koker et al. (2009), an Asp93Glu substitution, which also led to a complete loss of protein function. Moreover, the variant reported herein changes in the alanine at position 97 to an aspartate in the third helix of the TRP domain of the p67*^phox^* protein. Therefore, the variant described in our patient likely affects binding to GTPase RAC2 and the translocation of the p47*^phox^/*p67*^phox^* complex to the plasma membrane in activated neutrophils. Interestingly, other variants in the TRP domain of the p67*^phox^* protein have been previously reported in CGD patients who, as well, present a more severe clinical course (de Boer et al., 1994; Gentsch et al., 2010; Leusen et al., 1996; Martel et al., 2012; Patino et al., 1999).

**Table 2:** Published *NCF2* variants and the clinical phenotypes of patients with CGD

Publication	Nucleotide change (amino acid or mRNA change)	Age at diagnosis	Protein function	Clinical symptoms and outcome
El Kares et al. (2006)	c.1256A>T/c.1256A>T (p.Asn419Ile/p.Asn419Ile)	10 months	Not reported	CGD: not further detailed
	c.257+2T>C/ c.257+2T>C	19 months		
Kannengiesser et al. (2008)	c.364+1G>A/c.364+1G>A	Not reported	p67^null^ (Western blot)	CGD: recurrent life‐threatening bacterial and fungal infections
	c.364_366+2delGAGGT/ c.364_366+2delGAGGT			
	c.866_867delGT/c.866_867delGT (p.Val267Leufs*8/p.Val267Leufs*8)			
Koker et al. (2009)	c.229C>T/c.229C>T (p.Arg77*/p.Arg77*)	Not reported	p67^null^ (Western blot)	CGD: recurrent life‐threatening bacterial and fungal infections
	c.279C>G/c.279C>G (p.Asp93Glu/p.Asp93Glu)			
	c.304C>T/c.304C>T (p.Arg102*/p.Arg102*)			
	c.605C>T (p.Ala202Val/p.Ala202Val)		Residual oxidase activity	CGD: mild clinical manifestations
Bakri et al. (2009)	c.1169_1173del/ c.1169_1173del	9 years	p67^null^ (Western blot)	Multifocal osteomyelitis, bacteremia (*S. typhi*), hepatomegaly, brucellosis
		5 months		Peritonitis, hepatomegaly, perianal abscesses, lymphadenitis
		5 years		Granuloma in lung, died at age 6
		10 months		Died from sepsis (*Salmonella* spp.) at age 2, hepatomegaly
Gentsch et al. (2010)	c.366+2401_502del1380/0.366+2401_502del1380 (p.Val123_Trp167del/p.Val123_Trp167del)	Not reported	Inactive, unstable p67*^phox^*	CGD: recurrent life‐threatening bacterial and fungal infections
Teimourian et al. (2010)	(p.Met1_Lys58del/ p.Met1_Lys58del)	2 years	NADPH oxidase inactive	CGD: young age at presentation, severe disease
	c.196C>T/c196C>T (p.Arg66*/p.Arg66*)			
Badalzadeh et al. (2012)	c.304C>T/c.304C>T (p.Arg102*/p.Arg102*)	20 months	p67^null^ (Western blot)	CGD: BCG‐osis, recurrent pneumonia, hepatomegaly, hepatitis, and meningitis
	(p.Leu346fs*380/p.Leu346fs*380)	2 years		CGD: lymph node abscesses
		4.5 months		CGD: arthritis, severe diarrhea
	Δ exon 2/ Δ exon 2 (copy number variation)	7 months		CGD: pneumonia, pulmonary abscess, genital and perianal abscess, inguinal lymphadenopathy, hepatosplenomegaly
Martel et al. (2012)	c.29G>A/c.296G>A (p.Trp10*/p.Trp10*)	3 months	p67^null^ (Western blot)	CGD: severe and early presentation, splenic abscess, pneumonia
Roesler et al. (2012)	c.1000+2T>G/c.1000+2T>G	58 years	DHR test, residual NADPH oxidase activity	CGD: delayed onset, pulmonary aspergillosis, pulmonary fistula
		53 years		CGD: delayed onset, skin abscess, hepatic abscess, fungal pneumonia
Raptaki et al. (2013)	c.279C>G/c.279C>G (p.Asp93Glu/p.Asp93Glu)	1.8 years	p67^null^ (Western blot)	CGD: otitis media, candida, lymphadenitis
	c.502−1G>T/c.502−1G>T	2.5 years		CGD: pulmonary aspergillosis, salmonella gastroenteritis
	c.502−1G>T/c.502−1G>T	3 years		CGD: pulmonary aspergillosis, hepatic abscess, vertebral osteomyelitis, lymphadenitis, septicemia, deceased at age 14 years
Koker et al. (2013)	c.299C>T/c.299C>T (p.Arg77*/p.Arg77*)	<1, 2 years	p67^null^ (Western blot)	Chronic idiopathic thrombocytopenic purpura, Bechet disease, seborrheic dermatitis, stomatitis, gingivitis, and pericardial effusion. A total of three patients died from sepsis caused by *Aspergillus* infections in major organs (brain and lung)
	c.279C>G/c.366+1G>C/ c.279C>G/c.366+1G>C	4, 2, 3, <1, 17 years		
	c.304C>T/c.304C>T (p.Arg102*/p.Arg102*)	5 years		
	c.409T>A/ c.409T>A (p.Trp137Arg/ p.Trp137Arg)	2 years		
	c.410G>A/ c.410G>A (p.Trp137*/p.Trp137*)	5, <1 year		
	c.767_768dupAA/ c.767_768dupAA (p.Glu257Lysfs*15/ p.Glu257Lysfs*15)	1 years		
	c.605C>T/ c.605C>T (p.Ala202Val/ p.Ala202Val)	2 years	DHR residual oxidase activity	CGD: mild symptoms of Familial Mediterranean Fever and uveitis
Baba et al. (2014)	c.257+1G>A/ c.257+1G>A	5.5 years	p67^null^ (Western blot)	CGD: pneumonia, diarrhea, macrophage activation syndrome, bronchiectasis, lymphadenopathy, infections with *Salmonella* spp., *Aspergillus* spp.,
		0.67 years		Pneumonia, septicemia, deceased
		0.25 years		Lymphadenopathy, infections with *Klebsiella* spp., *Candida albicans,* and *Candida dubliniensis*
Roos et al. (2014)	c.605C>T/ c.605C>T (p.Ala202Val/ p.Ala202Val)	17 years	Residual oxidase activity	CGD: mild, recurrent abscess controlled by antibiotics, discoid lupus‐like rash, recurrent keratitis, conjunctival granulomata
		Diagnosed at birth		Recurrent oral ulceration, leg ulcers, folliculitis and skin abscesses, short period of diarrhea, and rectal bleeding
		8 years		Pustular and eczematous lesions of the scalp skin, recurrent chorioretinitis, severe uveitis
Chou et al. (2015)	c.1000+1G>A/ c.1000+1G>A	Adult	Residual oxidase activity	SLE: duodenitis, pulmonary infection
Ben‐Farhat et al. (2016)	c.257+2T>C/ c.257+2T>C	2–12 years (*n* = 11)	p67^null^ (Western blot)	CGD: recurrent life‐threatening bacterial and fungal infections. A total of 9 of 11 patients died mostly from respiratory illnesses
Wu et al. (2017)	c.550C>T/ c.550C>T (p.Arg184*/ p.Arg184*)	1.5 months	p67^null^ (Western blot)	Pneumonia, skin abscess, mycobacterial infections due to BGC
	c.137T>G/ c.137T>G (p.Met46Arg/ p.Met46Arg)			
	c.1130_1135delACATGG/ c.1130_1135delACATGG (p.Asp377 Met37del/ p.Asp377 Met37del)			
Vignesh et al. (2017)	(p.Thr279fs*/p.Thr279fs*)	Early childhood (*n* = 2)	p67^null^ (Western blot)	All three patients had colitis, suppurative lymphadenitis, failure to thrive, pneumonia Lung abscess in one patient (*Nocardia* spp.)
	c.1179–2A>T/c.1099C>T (p.Q367*)			
AlKhater (2019)	c.855_856del/ c.855_856del (p.Thr285fs*/ p.Thr285fs*)	3 years	DHR test, NADPH oxidase inactive	CGD: very‐early‐onset colitis, perianal abscesses (*E. coli* and *Klebsiella*) and fistula, juvenile idiopathic arthritis, failure to thrive

Many variants in the *NCF2* gene leading to CGD with a range in severity have been identified (Table 2) (AlKhater, 2019; Baba et al., 2014; Badalzadeh et al., 2012; Bakri et al., 2009; Ben‐Farhat et al., 2016; Chou et al., 2015; El Kares et al., 2006; Gentsch et al., 2010; Kannengiesser et al., 2008; Koker et al., 2009, 2013; Martel et al., 2012; Raptaki et al., 2013; Roesler et al., 2012; Roos et al., 2014; Teimourian, de Boer, & Roos, 2010; Vignesh et al., 2017; Wu, Wang, Zhang, & Chen, 2017). Part of this variability is due to the residual activity of the p67*^phox^* protein as observed in patients with an Ala202Val substitution (Koker et al., 2013; Roos et al., 2014) or in patients with a splice variant that deletes exons 11 and 12 (Roesler et al., 2012), all of which have a less severe form of CGD with a delayed onset compared with p67*^phox^* null mutations (Table 2). There have been reports that partially active p67*^phox^* is associated with an inflammatory phenotype (AlKhater, 2019; Chou et al., 2015; Muise et al., 2012). The gastrointestinal organs are most frequently affected by inflammation (Magnani et al., 2014; Rosenzweig, 2008), and noninfectious colitis is considered a common finding in CGD. Well‐defined immune‐mediated diseases are also reported in patients with CGD, such as systemic lupus erythematous, discoid lupus, and juvenile rheumatoid arthritis, among others (AlKhater, 2019; de Ravin et al., 2008). Various immunological mechanisms have been found to play a role in favoring the development of inflammation and granulomas in CGD patients (Petersen & Smith, 2013; Rosenzweig, 2008). Infection and inflammation appear to be the main driving forces for the granuloma formation observed in CGD patients (Conti et al., 2016; Petersen & Smith, 2013). Granuloma formation is the hallmark of CGD, hence the name, and consists of macrophage aggregates surrounding the organisms to prevent their spread in the host. This mechanism is particularly effective for containing mycobacterial infections (Petersen & Smith, 2013). Among those infections, BCG disease is the most frequently reported, accounting for 75% of all mycobacterial infections in CGD patients (Conti et al., 2016; Deffert et al., 2014). Most of the cases reported exhibit local or regional infection, with systemic disseminated disease accounting for 14% only of all BCG‐related infections in CGD patients (Deffert et al., 2014).

Regarding our patient, one important consideration is that she had a severe and an early presentation, which is often observed with *NCF2* variants that lead to no appreciable p67*^phox^* activity (Table 2). Nevertheless, some phenotypic variability is present even in this group of patients, which is most likely due to exposure to different pathogens in early life. In the case of our patient, the severe course and early onset may be explained by the early administration of the BCG vaccine at birth. The vaccine is routinely used for tuberculosis prevention in Saudi Arabia in a neonatal setting. The Saudi population has an estimated consanguineous marriage rate of 56%–60% (El Mouzan, Al Salloum, Al Herbish, Qurachi, & Al Omar, 2008). This practice exposes the Saudi Arabian population to a high risk of inherited diseases, including PID (Al‐Saud et al., 2015). Therefore, in such a setting, several important factors must be considered when administering the BCG vaccine, including the schedule, age at administration, family history of immune disorders, consanguinity, and the high risk for AR inherited diseases in the population. The vaccine should be withheld if there is any suspicion of an underlying immune deficiency. Alternatively, based on other previous reports in the region (Al‐Hammadi, Alsuwaidi, Alshamsi, Ghatasheh, & Souid, 2017; Al‐Saud et al., 2015), postponing BCG vaccination until 6–12 months of age with a special emphasis on excluding PID may be beneficial for reducing the risk caused by the use of live vaccines in these children (Bukhari et al., 2016). This crucial knowledge regarding the vaccine risks in certain populations should be available to clinical outreach and educational programs and in suburban areas, with a particular emphasis on the early detection of PID patients and their early care and management.

## CONFLICT OF INTEREST

The authors declare that they have no conflict of interest.

## AUTHOR CONTRIBUTIONS

SA performed clinical care of the patient and the literature review. CD, JC, and JB designed the study, performed the genetic analysis, and data interpretation. All authors contributed equally to the preparation and writing of the manuscript.
